# Antivirulence activity of azithromycin in *Pseudomonas aeruginosa*

**DOI:** 10.3389/fmicb.2014.00178

**Published:** 2014-04-22

**Authors:** Francesco Imperi, Livia Leoni, Paolo Visca

**Affiliations:** ^1^Pasteur Institute-Cenci Bolognetti Foundation and Department of Biology and Biotechnology “C. Darwin”, “Sapienza” University of RomeRome, Italy; ^2^Department of Sciences, “Roma Tre” UniversityRome, Italy

**Keywords:** antibiotic, cystic fibrosis, inflammation, macrolide, regulation, virulence

## Abstract

Antibiotics represent our bulwark to combat bacterial infections, but the spread of antibiotic resistance compromises their clinical efficacy. Alternatives to conventional antibiotics are urgently needed in order to complement the existing antibacterial arsenal. The macrolide antibiotic azithromycin (AZM) provides a paradigmatic example of an “unconventional” antibacterial drug. Besides its growth-inhibiting activity, AZM displays potent anti-inflammatory properties, as well as antivirulence activity on some intrinsically resistant bacteria, such as *Pseudomonas aeruginosa*. In this bacterium, the antivirulence activity of AZM mainly relies on its ability to interact with the ribosome, resulting in direct and/or indirect repression of specific subsets of genes involved in virulence, quorum sensing, biofilm formation, and intrinsic antibiotic resistance. Both clinical experience and clinical trials have shown the efficacy of AZM in the treatment of chronic pulmonary infections caused by *P. aeruginosa*. The aim of this review is to combine results from laboratory studies with evidence from clinical trials in order to unify the information on the *in vivo* mode of action of AZM in *P. aeruginosa* infection.

## INTRODUCTION

Antibiotics are used as first line drugs for the treatment of bacterial infections, but the widespread resistance to these agents combined with the shortage of novel antimicrobial compounds developed by the pharmaceutical industry results in an urgent need for new strategies to combat bacterial infections (Fernebro, 2011). Virulence factors are essential for bacterial pathogens to cause infection. Hence, suppression of virulence factor production, i.e., antivirulence therapy, has become an attractive anti-infective approach. In-depth understanding of the mechanisms by which pathogens cause disease has been essential for the recognition of suitable targets for antivirulence drugs (Cegelski et al., 2008; Rasko and Sperandio, 2010). Target-based rational design and screening of chemical libraries allowed the identification of a variety of virulence inhibitors (Clatworthy et al., 2007; Law et al., 2013). However, none of the antivirulence compounds developed so far have entered into clinical practice.

The unpredicted antivirulence activity observed *a posteriori* among macrolide antibiotics prompted revisiting of laboratory and clinical data to assess the potential of these compounds as antivirulence drugs. In this review, the clinical impact of azithromycin (AZM) on patients suffering from *Pseudomonas aeruginosa* infection are discussed in the light of the biological activities exerted by AZM on both the pathogen and the host.

## THE MULTIFARIOUS BIOLOGICAL ACTIVITIES OF MACROLIDES

**FIGURE 1:**
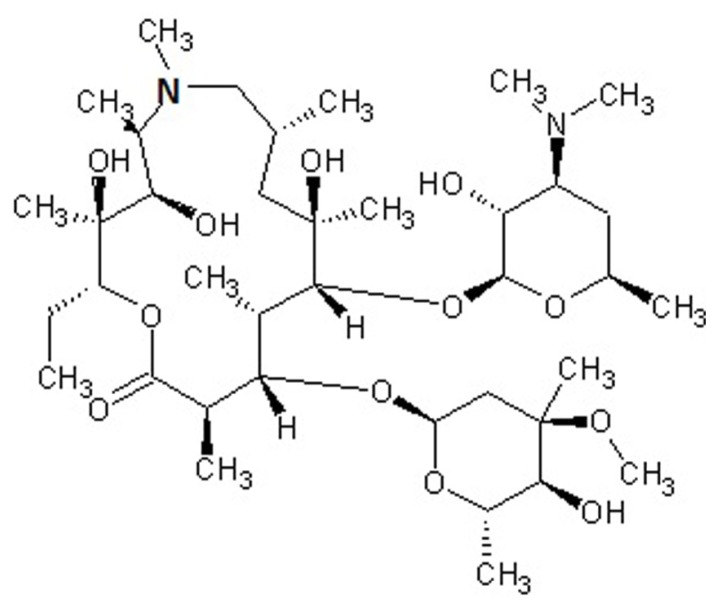
**
Chemical structure of azithromycin, a drug belonging to the azalide subclass of macrolides.** The 15-membered lactone ring is derived from erythromycin, upon incorporation of a methyl-substituted nitrogen atom (bold).

Macrolides are polyketide compounds characterized by the presence of a 14- (e.g., erythromycin), 15- (e.g., AZM, **Figure 1**), or 16- (e.g., josamycin) membered macrocyclic lactone to which one or more amino and/or neutral sugars are attached.

Macrolides have many important biological characteristics including antibacterial, antifungal and immunomodulatory properties. Erythromycin is the progenitor of this class of antibiotics and has served as the scaffold for the generation of newer semisynthetic macrolides (Washington and Wilson, 1985; Pal, 2006). AZM was launched in 1991 and rapidly became one of the most frequently used antimicrobials for outpatients (Hicks et al., 2013). A number of favorable pharmacological properties contributed to the success of AZM as an antibiotic, including acid resistance, a short time to achieve peak concentrations with an up to 800-fold accumulation in phagocytes at the infection site, and long half-life allowing a large single oral dose to maintain bacteriostatic activity in the infected tissue for 4 days (Girard et al., 1987; Foulds et al., 1990; Blumer, 2005).

Macrolide antibiotics inhibit bacterial growth by binding the 23S rRNA in the 50S subunit of the bacterial ribosome, thereby preventing the transfer of tRNA from the A to the P site of the ribosome. Binding to the A site prevents addition of an incoming amino acid-charged tRNA to the nascent polypeptide chain, ultimately aborting polypeptide growth (Retsema and Fu, 2001; Poehlsgaard and Douthwaite, 2005).

Some macrolides (e.g., rapamycin) lack antibacterial activity but possess potent immunosuppressive or immunomodulatory properties, and are therefore used in the therapy of autoimmune disorders and proliferative diseases. They act through different mechanisms at the level of the immune system, ultimately interfering with lymphocyte activation and cytokine production (McAlister et al., 2002; Ferrer et al., 2011; Salmond and Zamoyska, 2011). Starting from the late 1960s, evidence has been obtained showing that also macrolide antibiotics have anti-inflammatory and pro-kinetic effects which play a prominent role in some infections (Itkin and Menzel, 1970). These effects have extensively been reviewed in the recent literature (Amsden, 2005; Giamarellos-Bourboulis, 2008; Kanoh and Rubin, 2010; Steel et al., 2012; Aminov, 2013).

Macrolide antibiotics are typically bacteriostatic at therapeutic concentrations (Retsema et al., 1987). Different from cell-disrupting agents (e.g., β-lactams), they are unlikely to cause bacterial lysis and release of cell-associated pro-inflammatory molecules, thereby avoiding the induction of a detrimental inflammatory response (Spreer et al., 2003; Anderson et al., 2007). Sub-inhibitory concentrations of macrolides cause substantial inhibition of the synthesis of virulence factors in both Gram-positive and Gram-negative bacteria (Steel et al., 2012).

*Pseudomonas aeruginosa* is a paradigmatic example of a microorganism with intrinsic resistance to multiple classes of antibiotics, including macrolides. Nonetheless, a number of clinical studies have demonstrated that patients suffering from both intermittent and chronic *P. aeruginosa* infection, e.g., cystic fibrosis (CF), chronic obstructive pulmonary disease (COPD), and diffuse panbronchiolitis (DPB), benefit from AZM treatment (reviewed by Steel et al., 2012 and Aminov, 2013). Hereafter, the many effects of AZM on *P. aeruginosa* virulence and their impact on infection are discussed.

## EFFECT OF AZITHROMYCIN ON *P. aeruginosa* CELLS

The pathogenic potential of *P. aeruginosa* relies on the production of cell-surface components with pro-inflammatory and/or adhesion activity, and a huge arsenal of virulence factors (Driscoll et al., 2007). Moreover, its ability to adopt the biofilm lifestyle is critical in chronic infections. In *P. aeruginosa*, the extracellular polysaccharides (EPSs) Psl, Pel and alginate play an important role in maintaining the biofilm structure and in resistance to antibiotics and to the host immune system (Wei and Ma, 2013).

AZM is not approved for the treatment of infections caused by *P. aeruginosa* and there are no published breakpoints for this species. The AZM minimum inhibitory concentrations (MICs) for *P. aeruginosa* range from 8 to 512 μg/ml, depending on the strain and the testing procedure (e.g., Kita et al., 1991; Tateda et al., 1996; Nicolau et al., 1999; Morita et al., 2001). Early studies showed that sub-inhibitory AZM concentrations (sub-MIC AZM) suppressed motility and the production of several virulence factors, including proteases, pyocyanin, exotoxin A, phospholipase C (PLC), and EPSs in *P. aeruginosa* (Kita et al., 1991; Molinari et al., 1992; Molinari et al., 1993; Ichimiya et al., 1996; Nagino and Kobayashi, 1997; Favre-Bonté et al., 2003; Gillis and Iglewski, 2004). Since in *P. aeruginosa* the expression of many virulence factors is activated at the transcriptional level by the 3-oxo-C12-homoserine lactone (3OC12-HSL) and butyryl-homoserine lactone (C4-HSL) quorum sensing (QS) signal molecules, some studies focused on the effect of sub-MIC AZM on these two QS systems.

**FIGURE 2:**
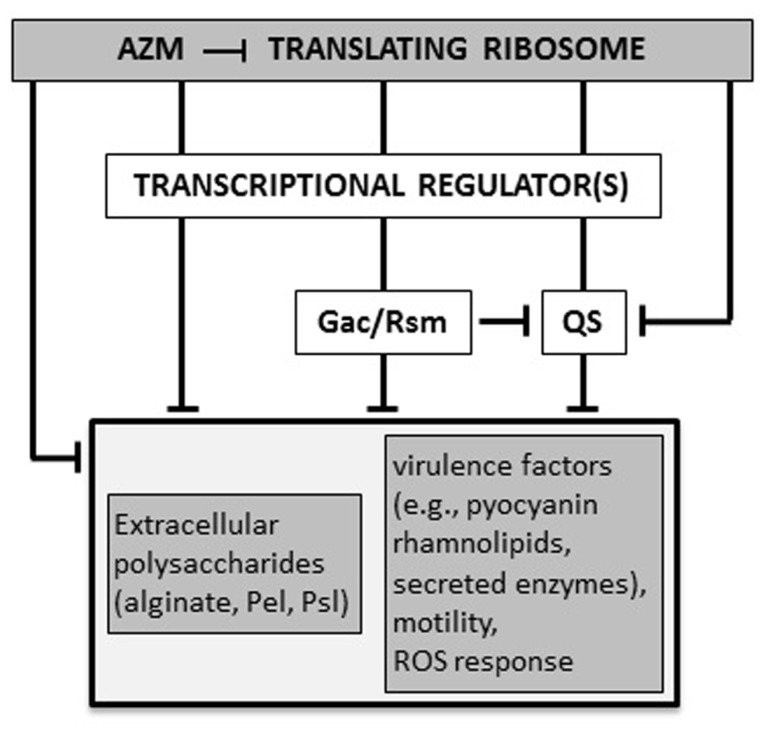
**
Proposed molecular mechanisms for AZM-mediated inhibition of *P. aeruginosa* virulence.** AZM, even at sub-inhibitory concentrations, interacts with the 50S ribosomal subunit and selectively affects the expression of a specific subset of genes, such as those for the Gac/Rsm and quorum sensing (QS) systems, and other unidentified transcriptional factors involved in regulation of virulence genes. Some genes (e.g., the QS regulator gene *rhlR*) may be affected by AZM via independent and overlapping regulatory pathways, both at the transcriptional and post-transcriptional level. The final outcome of this cascade is the suppression of a number of virulence-related phenotypes (see text for details). T-shaped lines represent negative controls.

AZM (2 μg/ml) reduces the production of both 3OC12-HSL and C4-HSL. Transcriptional repression of the corresponding synthase/receptor genes *lasI*/*lasR* and *rhlI*/*rhlR* contributes to this effect (Tateda et al., 2001). Accordingly, transcriptomic and proteomic analyses confirmed that AZM down-regulates the expression of many QS-dependent genes, as those encoding the pilus, flagellum, and oxidative stress response proteins (Nalca et al., 2006; Skindersoe et al., 2008; Kai et al., 2009). In* P. aeruginosa*, the AZM-affected transcriptome largely overlaps with the Gac/Rsm regulon, which also includes both *las* and *rhl* QS genes (Pérez-Martínez and Haas, 2011). In the Gac/Rsm regulatory pathway, the trans-membrane histidine kinase GacS phosphorylates the response regulator GacA in response to an unknown signal. Phosphorylated GacA activates the transcription of the two small regulatory RNAs (srRNAs) RsmY and RsmZ. At high concentrations, these srRNAs sequester the mRNA-binding protein RsmA, which acts as a translational repressor. RsmA directly or indirectly affects the expression of many virulence genes, including those implicated in QS regulation (Coggan and Wolfgang, 2012; Frangipani et al., 2014). Sub-MIC AZM reduced the expression of several genes in the Gac/Rsm regulon, and inhibited the transcription of the still uncharacterized ORFs PA0588–PA0584, which are required for full expression of *rsmZ* and *rsmY* (Kai et al., 2009; Pérez-Martínez and Haas, 2011). Therefore, AZM-dependent repression of 3OC12-HSL and C4-HSL synthesis could, at least in part, be explained by a cascade mechanism in which AZM represses expression of the PA0588–PA0584 genes which are required for full transcription of *rsmZ* and *rsmY*, resulting in down-regulation of QS gene expression. However, the effect of AZM on *rsmZ* and *rsmY* transcription was not completely abrogated in a PA0588–PA0584 deletion mutant, suggesting that AZM affects the Gac/Rsm system and QS also via alternative pathways (Pérez-Martínez and Haas, 2011; **Figure 2**). Allied to this, AZM also repressed transcription of genes for the synthesis of 3OC12-HSL and C4-HSL precursors (Kai et al., 2009).

Sub-MIC AZM has many pleiotropic effects on *P. aeruginosa* that cannot be explained only by its interference with the Gac/Rsm and QS systems. Examples are (i) the inhibitory effect on alginate production (Ichimiya et al., 1996; Favre-Bonté et al., 2003; Lutz et al., 2012), (ii) increased susceptibility to serum bactericidal activity, probably due to alterations of cell-surface structures such as lipopolysaccharides and outer membrane proteins (Tateda et al., 1993, 1994), (iii) increased susceptibility to some antimicrobials, due to down-regulation of the MexAB-OprM efflux pump (Sugimura et al., 2008), and (iv) killing of stationary-phase and biofilm-forming cells (Tateda et al., 1996; Imamura et al., 2005).

Some effects of sub-MIC AZM are dependent on a direct interaction with the ribosome. Indeed, heterologous expression of a *Clostridium perfrigens* 23S rRNA methylase gene in *P. aeruginosa* increased AZM resistance, counteracted inhibition of virulence factors production, and alleviated killing of stationary-phase cells (Kohler et al., 2007).

Macrolides block elongation of the nascent peptide chain and cause premature dissociation of the tRNA-charged growing polypeptide. Increased release of these abortive peptidyl-tRNAs, a phenomenon known as “drop-off”, impairs the normal turnover of tRNAs, affecting the overall protein translation rate (Retsema and Fu, 2001; Gödeke et al., 2013). Interestingly, overexpression in *P. aeruginosa* of the Pth peptidyl-tRNA hydrolase, an enzyme which releases uncharged tRNA from the peptidyl-tRNA, partially restored tRNAs turn-over and reversed some phenotypes caused by sub-MIC AZM, such as the stationary-phase killing and inhibition of pyocyanin and rhamnolipid production (Gödeke et al., 2013). Therefore, some phenotypes induced by sub-MIC AZM are determined by the increased peptidyl-tRNAs drop-off and defective turn-over of tRNAs. Since production of pyocyanin and rhamnolipids is dependent on the Rhl QS system, Gödeke et al. (2013) by analysing the *rhlI and rhlR* coding sequences found that the second codon of *rhlR* (AGG, encoding Arg) is very rarely used in *P. aeruginosa,* suggesting that translation of this gene could be particularly susceptible to defects in tRNAs turn-over. Accordingly, replacement of this codon with the preferentially used codon CGC reverted AZM-mediated inhibition of rhamnolipids and pyocyanin production (Gödeke et al., 2013). This result suggests that sub-MIC AZM may selectively affect the expression of distinct subset of genes, depending on their codon usage. Thus, the pleiotropic effects of sub-MIC AZM on *P. aeruginosa* are mainly due to AZM interaction with the ribosome and interference with protein synthesis. Differential codon usage in *P. aeruginosa* might explain the selective activity of AZM in translation of specific proteins. Besides RhlR, translation of still unidentified global regulators could also be affected by sub-MIC AZM concentrations, explaining AZM effects on *P. aeruginosa* transcriptome and physiology (Nalca et al., 2006; Skindersoe et al., 2008; Kai et al., 2009).

## EFFECT OF AZITHROMYCIN ON *P. aeruginosa* INFECTION

In Japan, macrolides have been used since the 1980s to treat DPB, a rare inflammatory lung disease that mainly affects elderly Asian people, in which chronic *P. aeruginosa* lung infection is associated with a poor outcome (Schultz, 2004). In the late 1990s, the similarities between DBP and CF drove Jaffé et al. (1998) to use AZM as a last resort agent for treatment of a teenager with CF on the waiting list for heart–lung transplantation; AZM treatment almost doubled the patient’s pulmonary function, leading to his removal from the list. This promising finding was confirmed by an open-label study on seven CF children infected by *P. aeruginosa* not responding to conventional therapy (Jaffé et al., 1998). Thereafter, several clinical trials have been conducted to validate AZM efficacy in CF. A recent meta-analysis of ten studies, including almost 1,000 patients, showed that AZM therapy is associated with a small but consistent improvement in respiratory function at 6 months, and has a good safety profile (Southern et al., 2012).

Hereafter, results from laboratory studies will be combined with evidence from clinical trials in order to summarize the information on the mode of action of AZM in *P. aeruginosa* infection. As described above, sub-MIC AZM exerts multiple effects on *P. aeruginosa*, including virulence inhibition, killing of stationary-phase and/or biofilm-forming cells, and synergism with other antimicrobials and with serum complement (Tateda et al., 1996; Imamura et al., 2005; Hoffmann et al., 2007; Lutz et al., 2012). In two chronic lung infection models of CF mice challenged with mucoid (alginate-producing) *P. aeruginosa* isolates, AZM suppressed QS-regulated virulence factors, drastically reduced the bacterial load in the lung, and improved lung pathology (Hoffmann et al., 2007; Tsai et al., 2009). However, in only one study AZM reduced *P. aeruginosa* associated mortality (Tsai et al., 2009). It was also found that AZM attenuated the inflammatory response and promoted macrophage phagocytic activity (Tsai et al., 2009), as previously reported for AZM-treated COPD patients (Hodge et al., 2008). The strongly reduced bacterial load in the lungs of AZM-treated mice could be explained by concomitant factors, including killing of biofilm-forming cells, improved phagocytic activity of macrophages, and/or increased susceptibility of QS-attenuated *P. aeruginosa* to the inflammatory/immune response (Hoffmann et al., 2007; Tsai et al., 2009). However, a reduced bacterial load was not observed for other antivirulence drugs capable of protecting mice from lethal *P. aeruginosa* lung infections (Miyairi et al., 2006; Imperi et al., 2013), suggesting that AZM suppresses the infection by targeting both *P. aeruginosa* and the immune system. Accordingly, relevant anti-inflammatory effects of AZM were also observed in CF mice that were not infected with *P. aeruginosa*, where AZM treatment resulted in attenuated cellular infiltration and reduced cytokine release (Legssyer et al., 2006). Therefore, the anti-inflammatory properties of AZM in the lung are also independent of its anti-*Pseudomonas* activity. It should be noted that previous work using non-CF murine models of lethal sepsis or pneumonia caused by non-mucoid and mucoid *P. aeruginosa* isolates, respectively, failed to show protective effects of AZM alone, although AZM acted synergistically with ceftazidime in both infection models (Nicolau et al., 1997, 1999). This suggests that experimental conditions have a considerable impact on the outcome of AZM treatment in animal infection models and/or that special features of CF lungs could contribute to improved AZM activity on *P. aeruginosa*.

The therapeutic efficacy of AZM in CF has been proven in many clinical trials. Beneficial effects were observed in CF patients chronically-infected with *P. aeruginosa* and, to a lesser extent, in uninfected CF patients (reviewed in Southern et al., 2012). The latter observation is consistent with the finding that AZM significantly reduced various serum inflammatory markers in CF patients not infected with *P. aeruginosa* (Ratjen et al., 2012), confirming again that AZM has anti-inflammatory effects independent of its antivirulence activity. However, some pulmonary function parameters, including forced expiratory volume in 1s (FEV1), were slightly less improved in patients without chronic *P. aeruginosa* infection compared to chronically-infected patients (Southern et al., 2012). Whether different outcomes are related primarily to the anti-*Pseudomonas* activity of AZM or to the early stage of lung disease in young patients uninfected with *P. aeruginosa* (Clement et al., 2006; Saiman et al., 2010) cannot be established at present.

Regarding the antivirulence activity of AZM in humans, a retrospective study observed a correlation between the inhibitory effect of AZM on PLC production by *P. aeruginosa* strains isolated from CF patients and the observed FEV1 improvement after AZM therapy (Nguyen et al., 2007), suggesting that *in vivo* PLC production is a main target of AZM. This finding fits well with the relevant role of *P. aeruginosa* PLC in the impairment of lung function in a mouse infection model (Wargo et al., 2011). A more recent study in intubated patients colonized with *P. aeruginosa* attempted to directly correlate the clinical effect of AZM on the patient with its antivirulence activity (van Delden et al., 2012). Although no relevant differences were observed between AZM-treated and untreated patients with regard to the occurrence of ventilator-associated pneumonia (VAP), a lower incidence of VAP was reported in a small sub-group (*n* = 5) of AZM-treated patients infected by *P. aeruginosa* strains producing high levels of rhamnolipids compared with the corresponding untreated group. Although preliminary, this observation suggests that AZM could be more effective in individuals infected by these highly virulent strains (van Delden et al., 2012).

Only few studies measured AZM levels during administration to CF patients. Data so far available suggest a wide range of AZM concentrations in sputa (0.6-79.3 μg/ml), depending on the individual patient and the dosing regimen (Baumann et al., 2004; Wilms et al., 2006). As discussed above, the growth inhibitory activity of AZM is strongly influenced by culture conditions. Since AZM MICs for *P. aeruginosa* are low (2–16 μg/ml) when determined in eukaryotic cell media or in mouse bronchoalveolar lavage fluid (Buyck et al., 2012), it may be possible that AZM also exerts some inhibition of *P. aeruginosa* growth in CF lungs. However, the insignificant differences in the frequency and concentration of *P. aeruginosa* in sputa from AZM-treated and untreated patients (Equi et al., 2002; Saiman et al., 2003; Clement et al., 2006) argue against this possibility.

## CONCLUSION

From a microbiological perspective, the therapeutic efficacy of an antimicrobial compound results mainly from its ability to impair bacterial growth, and this assumption has driven antibiotic research until now. AZM provides a clear example of how the therapeutic efficacy of an antimicrobial cannot exclusively be attributed to growth impairment. Anti-inflammatory and antivirulence properties likely predominate in the treatment of infections involving AZM-resistant pathogens, as in the case of *P. aeruginosa* pulmonary infections. Although there is strong evidence of antivirulence activity *in vitro*, it is not possible to assess the contribution of this activity to the efficacy of AZM *in vivo* because of concomitant anti-inflammatory activity and bactericidal effects under certain conditions. According to the current model, both antibacterial and antivirulence activities are based on the interaction of AZM with the ribosome, so that tightly interwoven effects on bacterial viability and production of virulence factors are hardly distinguishable* in vivo*.

Beneficial effects have so far been documented in CF patients treated with AZM for up to 6 months, while reduced efficacy was associated with longer treatment duration (Southern et al., 2012; Fleet et al., 2013). Loss of efficacy could be explained by the emergence of *P. aeruginosa* subpopulations that become insensitive to the antivirulence activity. This hypothesis could be verified by testing the virulence properties and the response to AZM in serial *P. aeruginosa* isolates collected during long-term AZM therapy. Under this condition, the emergence of macrolide resistance among both commensal bacteria and co-infecting pathogens is a matter of concern and deserves further study (Aminov, 2013).

In conclusion, AZM offers a unique model to reconsider the central dogma of antibiotic activity, but further research is needed to gain more insight into the effects of AZM on both the pathogen and the host.

## Conflict of Interest Statement

The authors declare that the research was conducted in the absence of any commercial or financial relationships that could be construed as a potential conflict of interest.
